# Counterfactual Thinking-Related Emotional Responses in Patients With Major Depressive Disorder

**DOI:** 10.3389/fpsyt.2020.589335

**Published:** 2021-01-08

**Authors:** Qi Zheng, Mei Liao, Bangshan Liu, WenWen Ou, WenTao Chen, Jin Liu, Yan Zhang

**Affiliations:** ^1^Department of Psychiatry, Xiamen Mental Health Center, Xiamen Xianyue Hospital, Fujian, China; ^2^Department of Psychiatry, The Second Xiangya Hospital, Central South University, Changsha, China; ^3^Hunan Key Laboratory of Psychiatry and Mental Health, China National Clinical Research Center on Mental Disorders (Xiangya), China National Technology Institute on Mental Disorders, Hunan Technology Institute of Psychiatry, Mental Health Institute of Central South University, Changsha, China

**Keywords:** major depressive disorder, counterfactual thinking, situation-focused, behavior-focused, emotion responses

## Abstract

**Objective:** To explore the emotional characteristics of counterfactual thinking (CT)-related emotion responses in patients with major depressive disorder (MDD) via the “counterfactual thinking gambling task (CTGT).”

**Method:** Twenty-five patients with MDD (the MDD group) and twenty-five healthy controls (the HC group) with matched demographic features were included. The 17-item Hamilton Depression Scale (HAMD) and the 14-item Hamilton Anxiety Rating Scale (HAMA) were used to assess the severity of depression and anxiety symptoms. The counterfactual thinking gambling task was applied to assess the situation-focused- and behavior-focused-CT-related emotion responses in the MDD group and the HC group.

**Results:** There was no significant difference in general demographic data between the two groups (*p* > 0.05). Compared with the HC group, the MDD group experienced higher levels of “*disappointment*” and lower levels of “*joy*” in the situation-focused CT paradigm (*p* < 0.05). However, the experience of “*regret*” and “*relief*” in the behavior-focused CT paradigm were not significantly different between the two groups (*p* > 0.05).

**Conclusions:** MDD is associated with an impaired situation-focused-CT-related emotion responses, and is often accompanied by increased disappointment and decreased joy; however, behavior-focused-CT-related emotion responses are not significantly affected in MDD. This pattern may represent the characteristic CT-related emotion responses of MDD.

## Introduction

According to some researchers, counterfactual thinking (CT) is a process of mentally generating alternatives to facts, with a key feature of an imagined better or worse counterpart of one's current status (1). CT helps people gain information about alternative possibilities and regulate their behaviors; it also helps to evoke creativity in complex situations, which is crucial in individual survival and ethnic adaptation (2). However, excessive CT may bring about negative effects, which is associated with increased risk for major depressive disorder (MDD) (3–6). Among the counterfactual thoughts, “*regret”* and “*relief* ,” and “*disappointment”* and “*joy*” are two pairs of emotional experience induced by different CT processes and are most commonly seen in MDD (7).

When a result is actually randomly assigned rather than in line with one's choice, individuals tend to compare different, randomly generated results of a particular situation. This is the basis of the *situation-focused CT* task (8), where the individual's choice does not affect the result. For example, when individuals are asked “What would have happened if the situation had been different?,” they may feel *disappointed* when the result fails to meet their expectations; and otherwise, they may feel *joyful*. At this moment, individuals mainly engages in causal reasoning by means of external attribution, assuming that the unexpected results are not caused by their own choice but are more influenced by environmental or other factors.

When a result is associated with one's own choice, individuals tend to compare the outcomes resulted from different choices. This is the basis of *behavior-focused CT* task (9), where individuals' choices of different behaviors lead to different results. For example, when individuals are asked “If I make a different choice, what would the outcome be?,” they may experience *regret* if their imagined, alternative choice could lead to a better result than that caused by their own choice; and otherwise, individuals may experience *relief* . In this case, individuals usually engage in causal reasoning by means of internal attribution and connect the adverse result to their own influence.

Although there have been many studies on CT in patients with MDD to date, the characteristics of CT-related emotion responses in MDD are still unclear. Some studies suggested that the severity of symptoms of depression is positively correlated with the likelihood of excessive CT, which may contribute to the persistence or aggravation of the symptoms (3, 10). Patients with MDD may be overwhelmed by their “painful past” and unable to put it aside, which may lead to stronger negative emotions such as *regret* and *disappointment* (11). However, some other studies have come to the opposite conclusion that CT in MDD might be suppressed (4, 12). In a study by Chase et al. patients with MDD had reduced *regret* that was proportional to their score of a self-reported apathy scale (13), indicating that MDD was associated with subdued CT. The authors suggested that those with MDD might consider themselves to be in a desperate situation or might lack interest in the reality, which may inhibit their tendency to think counterfactually.

These inconsistent results may be partly attributed to methodological issues. Most previous studies used self-report questionnaires, as well as story-telling and comic book methods (14, 15). These methods have great limitations. Firstly, the scenario settings of the above methods are mostly based on patients' past experience or simulated imagination, i.e., their experience are not simultaneous and instantaneous. Secondly, both the self-report questionnaire and story-telling methods evaluate CT through retrospective questions, which may introduce considerable recall biases. Lastly, previous studies paid little attention to different CT processes, such as situation-focused CT and behavior-focused CT, and did not conducted quantitative analyses of the generated emotions. Considering these limitations, the application of new methods assessing different CT processes and related emotion responses instantaneously is urgently needed.

Recently, researchers have adopted a new psychological experimental paradigm, namely “the counterfactual thinking gambling task (CTGT)” (16, 17), to assess different CT processes and their related emotions. The task requires participants to make a choice with the goal of winning as much money as possible and then rate their emotions for the winning or losing. The task provides data based on the results of the participant's choices as well as the results of their alternative choices. The participants are required to provide feedback on their emotions generated from two different results, one dependent on their actual choice and the other associated with “the alternative choice” (producing situation-focused and behavior-focused CT, respectively). This paradigm is able to provide situations to trigger real time CT processes. The CTGT overcomes the limitations of previous methods and is a useful method to assess CT-related emotion responses quantitatively. This task has been widely used in patients with obsessive-compulsive disorder (17), autism (18), psychiatric symptoms (19), and trait anxiety (20, 21), and helped to generate valuable information on the characteristics of CT-related emotion responses regarding these disorders. However, it has yet been applied to the assessment of patients with MDD.

Here, by reporting our study on CT-related emotion responses in patients with MDD using CTGT, we aim to explore the relation between emotion responses and MDD in situation-focused and behavior-focused CT processes. Since patients with MDD usually show excessive negative emotions, we made a hypothesis that patients with MDD might experience amplified negative emotions (such as *disappointment* and *regret*) regarding their decision-making results under different CT processes, and have weakened positive emotions (such as *joy* and *relief*).

## Materials and Methods

### Participants

Twenty five patients with MDD who were hospitalized or treated in the outpatient clinic at the second Xiangya Hospital of Central South University were enrolled from october 2018 to February 2019. The diagnostic interview was conducted using the patient version of DSM-IV (SCID) for axis i disorder for the screening of HCs and patients with MDD. All the MDD patients were in an acute episode at that moment. participants meeting the criteria of other psychiatric disorders in the SCID interview were excluded.

#### Major Depressive Disorder (MDD)

The inclusion criteria of the MDD group are as follows: (1) 18–50 years of age, male or female, and Han ethnicity; (2) meet the diagnostic criteria provided in the Structured Clinical Interview for DSM-IV (SCID); (3) have not taken any antidepressants for at least 2 weeks; (4) have a total score >17 in the 17-item Hamilton Depression Scale (HAMD); (5) with an education level of primary school and above; and (6) right-handed.

The exclusion criteria for the MDD group were: (1) with histories of epilepsy, craniocerebral trauma or other organic diseases; (2) with histories of drug or substance abuse in the past six months; (3) have severe physical comorbidities; (4) at great risk for suicide (diagnosed with the MINI6.0.0B module); (5) pregnant or lactating women; (6) have undergone electroconvulsive therapy or transcranial magnetic stimulation therapy in the past 3 months; or (7) have a lifetime history of vagus nerve stimulation or deep brain stimulation treatment.

#### Healthy Controls (HCs)

A total of 25 HCs were enrolled from October 2018 to February 2019, with the inclusion criteria of (1) 18–50 years of age, male or female, and Han ethnicity; (2) have a total HAMD score of below 7; (3) have an education level of primary school or above; (4) right-handed; (5) with good mental condition and no history of mental disorders or family history of mental disorders; and (6) have no history of psychotropic treatment, including antidepressants, mood stabilizers, antipsychotics, and anticonvulsants.

This study was approved by the Medical Ethics Committee of the Second Xiangya Hospital of Central South University. All the participants provided written informed consent form before the initiation of experiment.

### Demographic and Clinical Data Collection

General demographic data (age, gender, education, marital status, medication status) were collected from all the included participants using a self-designed questionnaire. The HAMD and the 14-item Hamilton Anxiety Rating Scale (HAMA) were used to assess their mental conditions.

### Experimental Task Paradigm: The Counterfactual Thinking Gambling Task (CTGT)

**Figure 1:**
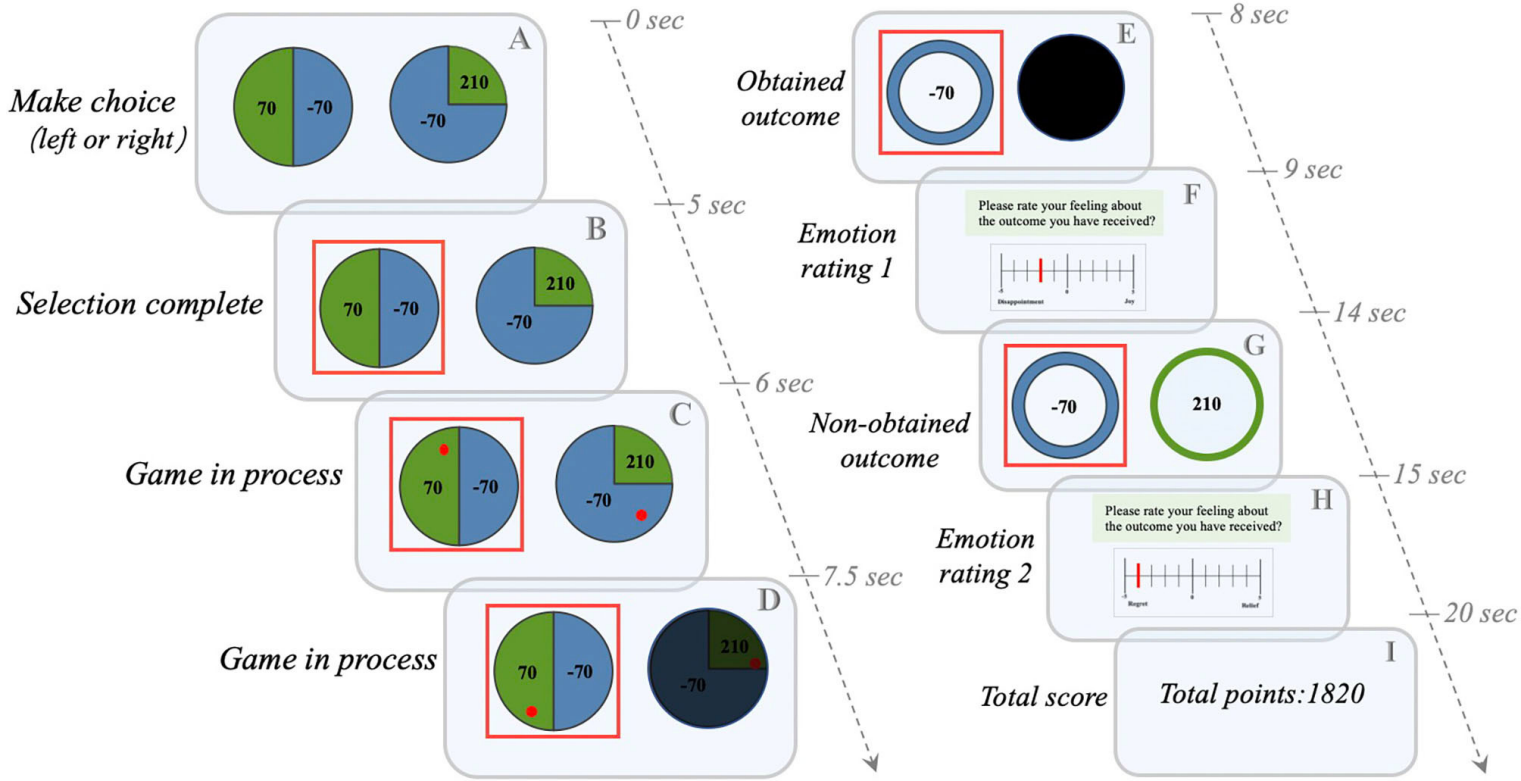
The Counterfactual Thinking Gambling Task (CTGT). The gambling task used in the Gillan et al. study (17) was modified. (1) The participant chooses between two “wheels of fortune” **(A)**. For example, if the subject chooses the left wheel, he/she may win (green) or lose (blue) 70 points with a chance of 50%; if he/she chooses the right wheel, he/she may win 210 points with a chance of 25% (Green) or lose 70 points with a chance of 75% (blue). (2) After the participant completes the selection, the selected wheel will be marked with a red box **(B)**. (3) The red lucky ball starts to rotate in the two wheels, and the unselected wheels are gradually covered with black color **(C,D)**. (4) When the ball stops rotating, only the outcome of the selected wheel is provided (e.g., losing 70 points), while the result of the unselected wheel is completely covered with black color **(E)**. (5) The participant is asked to provide the first emotional rating of the obtained results, ranging from−5 (extremely negative) to 0 (neither positive nor negative) to 5 (extremely positive) **(F)**. (6) After the rating, the result of the unselected wheel is provided (e.g., winning 210 points) **(G)**. (7) The participant is asked to provide the second emotional rating after comparing the obtained outcome with the results of unselected wheels **(H)**. (8) The total score for each round of experiments is calculated (e.g., the total score is 1820 points) **(I)**.

The experimental task paradigm (Figure 1) used in this study was adapted from the study of Gillan et al. (17) and compiled by the psychology software Eprime 2.0. The task includes 80 trials, which takes ~25 min to complete. Before the beginning of the task, the participants were told to make a choice between two wheels to earn a best possible score, which represents the cash reward they could receive in the end. In each trial, participants are required to choose between two wheels displayed on a computer screen; and each choice includes a combination of a value (from 4 possible values: −210, −70, +70, +210) and a set of probability (from 2 sets: 0.5/0.5 and 0.25/0.75). The area where the lucky red ball settles after the wheel rotation determined the score of the trial.

In the next step, two screens are presented to the participant. The first screen shows only the result of the selected wheel, and the final score depends on where the lucky ball stops randomly. This screen is designed to induce a “*situation-focused CT*” process, i.e., “What would happen to the score if the lucky ball had landed elsewhere on the wheel?” The participant then rate their emotion (from −5 [extremely negative] to 0 [neither positive nor negative] to 5 [extremely positive]) for the first time by comparing the difference between the obtained result (the area where the lucky ball landed) and the non-obtained result (the area where the lucky ball did not land). When the lucky ball stops in an area with a higher value, meaning the result is in accordance with the expectation, the participants may experience *joy*. In contrast, when the lucky ball lands in the area with a lower value, indicating that the result does not meet the expectation, the participants may experience *disappointment*. The second screen further displays the result of the unselected wheel, which is designed to induce a “*behavior-focused CT*” process, i.e., “What would happen to the score if I chose another wheel?” The second rate of emotion is based on the comparison of scores of the selected and unselected wheels. If the score of the selected wheel is higher than that of the unselected wheel, meaning the participants have made the correct decision, the participants may experience *relief* . In contrast, when the score of the selected wheel is lower than that of the unselected wheel, meaning the participants have made the wrong choice, the participants will be more likely to experience *regret*.

### Statistical Analysis

Data analysis was performed using the software SPSS Version 22. The sociodemographic and behavioral characteristics were assessed via appropriate bivariate statistical tests. The demographic variables (including age, education level and the total scores of HAMD and HAMA) of this study showed skewed distribution. The data of positive emotional ratings (“*joy*” and “*relief* ”) were normally distributed and the distribution of the negative emotional ratings (“*disappointment*” and “*regret*”) was skewed. Normally distributed continuous data were analyzed using *t*-test and Pearson's correlation analysis; continuous variables showing skewed distribution were analyzed using Mann-Whitney test and Spearman's correlation analysis. For all these analyses, the two-tailed significance level was set at 0.05.

## Results

### Demographic and Clinical Data for the MDD and HC Groups

As shown in Table 1, there were no significantly different demographic parameters between the MDD group and the control group (all *P* > 0.05). The total scores of HAMD and HAMA were significantly higher in patients with MDD than HCs (*P* < 0.05).

**Table 1:** Demographic and clinical characteristics of MDD patients and healthy controls.

	**MDD (*N* = 25)**	**HC (*N* = 25)**	**x^**2**^/z**	***p***
**Demographics**				
Age	25.8 ± 1.37	23.92 ± 1.30	−0.927	0.354
Gender (F [%])	17 (68.00)	20 (80.00)	0.936	0.333
Education (y)	14.12 ± 0.60	14.72 ± 0.56	−0.857	0.392
**Marital status**				
Single *N* (%)	14 (56.00)	21 (84.00)	4.971	0.083
Married *N* (%)	10 (40.00)	4 (16.00)		
Divorced *N* (%)	1 (4.00)	-		
**Scale evaluation**				
HAMD	21.60 ± 0.80	0.56 ± 0.22	−6.196	** <0.001**
HAMA	10.16 ± 0.98	1.12 ± 0.38	−5.815	** <0.001**

*Values in bold font indicate statistical significance. MDD, major depressive disorder; HC, healthy control; HAMD, the 17-item Hamilton Depression Scale; HAMA, the 14-item Hamilton Anxiety Rating Scale*.

### CT-Related Emotion Responses

#### Situation-Focused-CT-Related Emotion Responses

**Figure 2:**
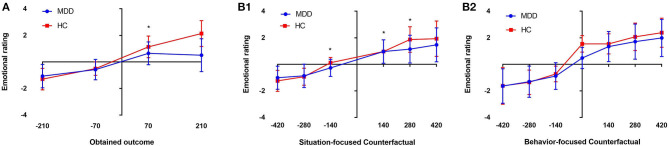
Mean emotion ratings for the four obtained outcomes (−210/−70 and +70/+210). The MDD patients showed weaker positive responses to wins (+70) than the healthy controls (HCs) (*p < 0.05, **A**). **(B1)** Shows the situation-focused CT (obtained outcome—unobtained outcome from that wheel) emotion responses for the first rating, followed by the comparison **(B1)**. **(B2)** Shows the behavior-focused CT (obtained outcome—outcome from the unselected wheel) emotion responses for the second rating, followed by a comparison between choices **(B2)** (*p < 0.05; NS, non-significant). The emotion scale ranged from−5 (extremely negative) to +5 (extremely positive).

**Figure 3:**
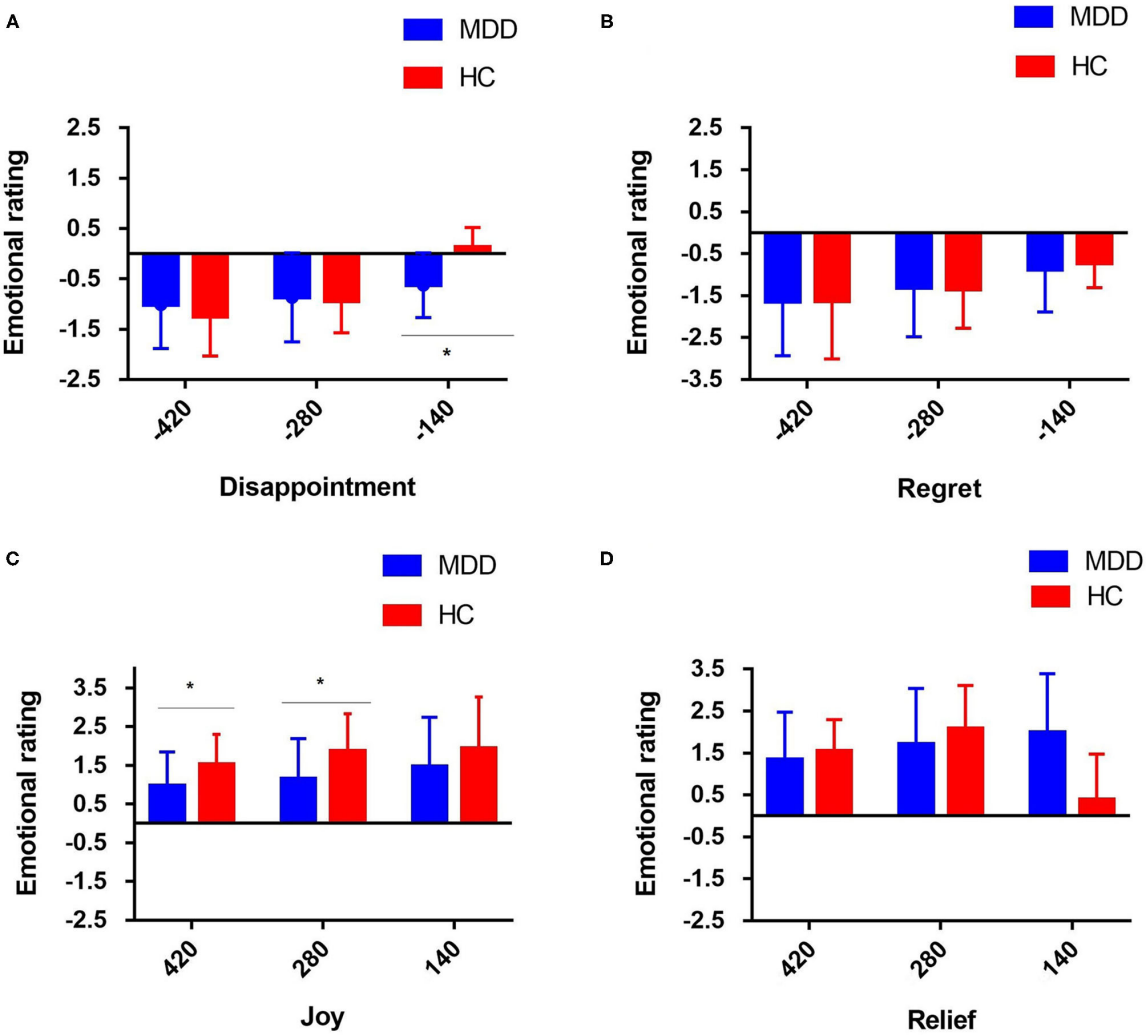
Mean individual emotion rating for the four different events (note: *disappointment* and *joy* refer to the intra-option comparison in the situation-focused CT, while *regret* and *relief* refer to the inter-choice comparison in the behavior-focused CT for the two groups of participants. For situation-focused CT, the participant could experience *disappointment* **(A)** in the case of relative loss, i.e., the obtained outcome is worse than the non-obtained outcome of the selected wheel, or *joy* **(C)** in the case of relative gain, i.e., the obtained outcome is better than the non-obtained outcome of the selected wheel. For example, intra-option comparison: loosing 70 when they could have lost 210 was classified as a relative gain, and winning 70 when they could have won 210 was classified as a relative loss. For the behavior-focused CT, information on the outcome of the unselected wheel is available, and the participant could experience *regret* **(B)** when the obtained outcome is worse than that of the unselected wheel or *relief* **(D)** when the obtained outcome is better than that of the unselected wheel (i.e., inter-choice comparison) (*p < 0.05. The emotion scale ranged from−5 [extremely negative] to +5 [extremely positive]).

Through this experiment, we have found that the absolute value of participants' emotional rating was positively correlated with the values of their winning or losing. In addition, the positive mood score of patients with MDD increased significantly when they earned money (*P* < 0.05) (Figure 2A). For the first emotional rating, the participants could experience *disappointment* if the result does not meet their expectation, i.e., the value the actually obtained is lower than the non-realized outcome, or *joy*, if the result is in accordance with the expectation, i.e., their obtained value is higher than the non-realized outcome. There was also a significant difference in the emotion response caused by comparing the obtained results and the non-obtained results for the same wheel (*situation-focused CT*: “what could have they won had the ball landed elsewhere?”) between the two groups (*P* < 0.05) (Figure 2B1). The *disappointment* induced by *situation-focused CT* was significantly increased in the patients with MDD, compared with the HCs (*P* < 0.05) (Figure 3A), but the *joy* was significantly decreased (*P* < 0.05) (Figure 3C). In summary, this study revealed that when the results met their expectations, patients with MDD showed weaker *joy* than HCs. And when the gap between the reality and expectation was small, those with MDD were significantly more likely to report *disappointment*.

#### Behavior-Focused-CT-Related Emotion Responses

For the second emotional rating, information about the outcome of the unselected wheel is available, i.e., the participants are shown what they would have won/lost had they acted differently. For the *behavior-focused CT* (“What would happen to the score if I chose another wheel?”), the participant can experience *regret* when the obtained outcome is worse than that on the unselected wheel, or *relief* , when the obtained outcome is better than that on the unselected wheel. However, there was no significant difference in emotion ratings that influenced by the *behavior-focused CT* between the two groups (*P* > 0.05) (Figure 2B2). Regardless of the comparison between the obtained and non-obtained outcomes and whether the outcome of the non-selected wheel was positive or negative, the patients with MDD did not show obvious *regret* (*P* > 0.05) (Figure 3B) or *relief* (*P* > 0.05), compared with the HC group (Figure 3D).

### The Association Between Depression and CT-Related Emotion Responses

As shown in Table 2, no significant correlation was found between the severity of depression symptoms and the situation/behavior-focused CT emotion reactions (*P* > 0.05).

**Table 2:** Correlations between depression symptoms and situation/behavior-focused CT emotion reactions (r).

	**HAMD(*r*)**
**Disappointment**	
Situation-focused CT (−420)	−0.163
Situation-focused CT (−280)	−0.080
Situation-focused CT (−140)	0.074
**Joy**	
Situation-focused CT (140)	0.039
Situation-focused CT (280)	0.007
Situation-focused CT (420)	0.067
**Regret**	
Behavior-focused CT (−420)	−0.068
Behavior-focused CT (−280)	−0.085
Behavior-focused CT (−140)	−0.014
**Relief**	
Behavior-focused CT (140)	0.096
Behavior-focused CT (280)	0.088
Behavior-focused CT (420)	0.099

*HAMD, the 17-item Hamilton Depression Scale*.

## Discussion

To our knowledge, this study used the psychological paradigm of the CTGT for the first time to explore the characteristics of CT-related emotion responses in MDD. The results showed that MDD patients exhibited impaired emotion responses in *situation-focused CT* processes, which is manifested as increased *disappointment* and decreased *joy*, while the emotion responses in *behavior-focused CT* processes remained relatively intact in MDD, manifested as relatively normal experience of *regret* and *relief* . In contrast to our hypothesis, patients with MDD did not exhibit pervasive increased negative and decreased positive emotions, but showed selective alterations in situation-focused-CT-related emotion responses. We propose that the impaired emotion responses in *situation-focused CT* processes and preserved emotion responses in *behavior-focused CT* processes may serve as a characteristic CT processing in MDD.

### Impaired Situation-Focused-CT-Related Emotion Responses in MDD

It is well-known that some patients with MDD have anhedonia as their primary symptom, and there is a general decline in their positive emotional experience (22). Thus, they do not experience as much joy when expectations are met (23, 24). In particular, when an actual result is slightly higher than the expected value, the MDD patients did not exhibit much sensitivity to the positive stimuli, resulting in their inability to experience the same degree of positive emotions. The lower sensitivity may be a feature of depressive state, or may be the cause of an increased risk for depression. “*Disappointment*” is another emotion experience based on situation-based counterfactual thinking, which is considered a sense of powerlessness in the face of the outcome. MDD is more likely to cause obvious disappointment when the individual is faced with unchangeable situations. This suggests that patients with MDD cannot separate their emotion responses from facts and have excessive emotion responses to events that cannot be influenced by themselves, indicating that MDD patients may have a more field-dependent cognitive style, which is consistent with previous studies (25). This field-dependent cognitive style can lead to patients' inability to distinguish between situations that they can and cannot influence, leading to excessive negative emotions, and higher risk for depression.

### Preserved Behavior-Focused-CT-Related Emotion Responses in MDD

In the situation where the patients thought they could exert influence, their behavior-based CT-related emotion responses are relatively intact, and there was no significant difference between their emotion response and that of the HCs. This is inconsistent with the clinical phenomenon we usually observe, i.e., most patients with MDD are more likely to blame themselves and feel guilty, as well as exaggerate their responsibility in an event. They may attribute negative outcomes to themselves, especially when the someone close to them are involved, e.g., they may feel that they are a burden of their family or friends. However, in this experiment, the participants are well-aware that this was just an experiment, and the results of the experiment have no impact on their real life (except for receiving some rewards); thus, the impact on their own behavior did not result in strong emotions. This is also different from the *situation-oriented CT*, which is more similar to real life situations, as participants are more powerless to the result. Therefore, this experiment may not be able to simulate real life situations that are closely related to emotions of patients with MDD, which might be the reason for the insignificant differences between the two groups.

### Clinical Implications

In clinical settings, most patients with MDD have predisposing factors. Thus, it is crucial to understand how MDD patients produce CT about negative events. Studies have found that patients with severe depression are more likely to engage in CT than those with mild to moderate depression (3, 26, 27). One possible reason is that patients with MDD have negative cognitive biases that make them think about the “painful past” repeatedly, which brings about more negative emotions. Thus, a vicious circle is formed, strengthening the relationship between depression and CT (11). Niedenthal et al. (28) found that CT may also be related to other emotions, such as shame and guilt. In the natural follow-up study, we will focus on the characteristics of CT across different stages of MDD, such as high-risk period, prodromal period, remission period, relapse period, among others, to explore whether altered CT are state-dependent or trait-like characteristics of MDD.

According to some studies, adult men in prison and patients with autism showed lower levels of *regret* (18, 19). However, patients with obsessive-compulsive disorder had stronger feelings of *regret* and *disappointment*, which may be associated with compulsive rumination (17). We infer that this may be a specific emotion feature of MDD and one of the indicators that distinguishes MDD patients from patients with other mental disorders. In addition, previous studies using self-report questionnaires, story-telling and other methods to investigate the relationship between the intensity of CT and emotions found that patients with MDD exhibited more counterfactual thoughts and less rationality (3, 15). Marian et al. (29) found that the severity of depressive symptoms was negatively correlated with the intensity of CT in patients with prefrontal cortex damage. However, in our study, the intensity of CT is constant in different subjects, as each trial produces one incidence of CT. Thus, we are unable to infer whether patients with MDD are associated with more excessive CT than HCs. Future studies may address this issue by combining experimental tasks and self-report questionnaires to comprehensively investigate the intensity of CT and CT-related emotions and behaviors in patients with MDD.

## Limitations

To our knowledge, the present study is the first one to use objective behavioral data to investigate CT-related emotion responses in patients with MDD. Despite the exploratory nature and innovation of this study, several limitations should be noted. Firstly, because of the relatively small sample size of this study, it was unable for us to conduct subgroup analyses to investigate the effects of different episodes (first-episode vs. recurrent), severity (moderate vs. severe vs. very severe), medication (medication-free vs. medicated), and comorbidity (comorbid with other psychiatric disorders vs. comorbidity-free) of MDD. Secondly, this study did not include a follow-up survey of MDD, thus, it remains unclear whether the impaired situation-focused-CT-related emotion responses (increased *disappointment* and decreased *joy*) exist in different stages of MDD, such as the preonset stage and remission stage. Thirdly, the self-rating of emotions is subjective and transient, as we did not monitor the objective indicators of emotion arousal, such as skin-conductance or cortisone level, during the trial. Thus, it is unknown whether the self-reported emotions is accurate and meaningful enough for clinical practice.

## Conclusions

In this study, we first explored the characteristics of CT-related emotion responses in MDD via CTGT. Patients with MDD showed impaired situation-focused CT-related and preserved behavior-focused emotion responses, leading to increased disappointment, decreased joy, and relatively intact regret and relief experience. This pattern may represent the characteristic CT-related emotion responses of MDD. It also calls for special attention to psychological interventions of situation-focused CT in MDD. Future studies are needed to explore whether this pattern exists in different stages of MDD and whether it is unique to MDD or shared among different mental disorders, such as schizophrenia and bipolar disorder.

## Data Availability Statement

The raw data supporting the conclusions of this article will be made available by the authors, without undue reservation.

## Ethics Statement

The studies involving human participants were reviewed and approved by the Medical Ethics Committee of the Second Xiangya Hospital of Central South University. The patients/participants provided their written informed consent to participate in this study.

## Author Contributions

QZ: collected data, drafted the manuscript, edited, and submitted the manuscript. ML: conducted statistical analysis and critically revised the manuscript. WO and WC: collected data. BL and YZ: conceptualized and designed the study. JL: conceptualized and designed the study, critically reviewed, and revised the manuscript. All authors have approved the final version of this manuscript.

## Conflict of Interest

The authors declare that the research was conducted in the absence of any commercial or financial relationships that could be construed as a potential conflict of interest.
